# Untargeted Chemical Profiling of Two-Dimensional Gas Chromatography Coupled with High-Resolution Mass Spectrometry Data for Botrytized Wines via Topological Data Analysis

**DOI:** 10.1021/acs.analchem.5c05342

**Published:** 2025-11-14

**Authors:** Nemanja Koljančić, Seol Ah Park, Davide Gurnari, Paweł Dłotko, Jooyoung Hahn, Ivan Špánik

**Affiliations:** † Institute of Analytical Chemistry, Faculty of Chemical and Food Technology, 201272 Slovak University of Technology in Bratislava, Radlinského 9, 812 37 Bratislava, Slovak Republic; ‡ Department of Mathematics and Descriptive Geometry, Faculty of Civil Engineering, 377063 Slovak University of Technology in Bratislava, Radlinského 11, 810 05 Bratislava, Slovak Republic; § Dioscuri Centre in Topological Data Analysis, Institute of Mathematics, 49559 Polish Academy of Sciences, 8 Śniadeckich Street, 00-656 Warsaw, Poland; ∥ Department of Software Engineering, Faculty of Nuclear Sciences and Physical Engineering, 679861 Czech Technical University in Prague, Trojanova 13, 120 00 Praha 2, Czech Republic

## Abstract

Advanced chemical profiling of complex samples, such as botrytized wines, requires advanced analytical techniques capable of capturing subtle compositional variations. In this study, we introduce a statistically robust framework that leverages a topological data analysis (TDA) tool, Ball Mapper, in the context of comprehensive two-dimensional gas chromatography (GC × GC) with high-resolution time-of-flight mass spectrometry (HR-TOF-MS) to obtain untargeted identification of sample-specific chemical markers. A key design element of the proposed approach is its ability to numerically process the immense data volume generated per sample, whose statistical and chemical significance is often difficult to interpret using conventional methods. Each of the 34 wine samples yielded over 470,000 mass spectral functions, which were discretized, normalized, and clustered to obtain representative and relatively unique discrete mass spectral vectors in high-dimensional space. With only two interpretative parameters, the proposed framework uncovered 2,792 extracted mass spectral distributions, from which 1191 discriminative features were identified, including 334 compounds assigned to known volatile organic compound classes. The resulting chemical signatures reflected regional differences in fermentation style, grape variety, botrytization conditions, and microbial activity. Moreover, statistically robust framework of using Ball Mapper revealed consistent grouping patterns both within and between wines. These findings demonstrate that the proposed framework can support chemical characterization complex natural matrices and serve as a general strategy for analyzing any domains where GC × GC with HR-TOF-MS data are collected.

## Graphical abstract

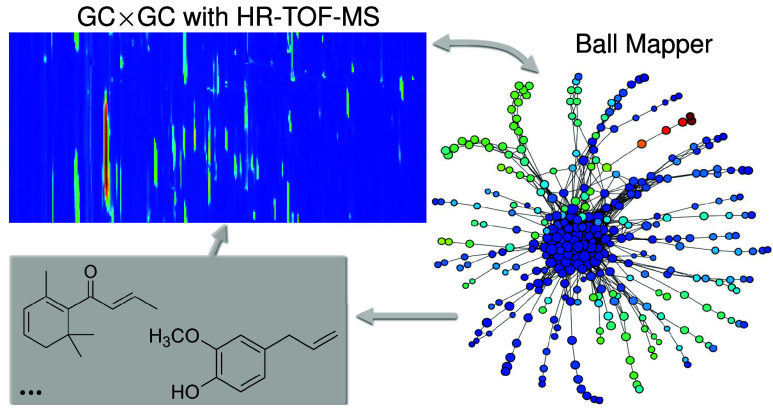

## 1 Introduction

Advanced analysis of complex chemical mixtures, such as biological fluids, environmental samples, and fermented beverages, often relies on comprehensive two-dimensional gas chromatography (GC × GC) coupled with high-resolution time-of-flight mass spectrometry (HR-TOF-MS).^1–3^ This powerful analytical platform offers exceptional separation capacity and high spectral resolution, enabling the detection of tens of thousands of ion signals per sample. However, the immense volume and high dimensionality of GC × GC with HR-TOF-MS data, compounded by baseline noise, overlapping peaks, and variation in signal intensities, pose significant challenges for interpretation, particularly in untargeted workflows where relevant features are not known a priori. To address these challenges, we introduce a computational framework for untargeted identification of sample-specific mass spectra in GC × GC with HR-TOF-MS data, based on Ball Mapper which is a topological data analysis (TDA) tool.^4,5^ As a case study, we apply the proposed method to a data set of botrytized wines, sweet wines produced from grapes infected by the noble rot fungus *Botrytis cinerea*. During botrytization, the metabolic transformation of grapes profoundly alters the volatile organic compound (VOC) profile,^6^ making these wines a chemically rich and compositionally dynamic model system for evaluating advanced analytical strategies.

A common visual representation of GC × GC for each modulation period is rasterization, which organizes detector events into pixelated columns aligned along the *x*-axis (first-dimension separation time) and *y*-axis (second-dimension separation time).^7,8^ Although the representation is informative, the process of extracting meaningful features for classification, authentication, or quantitative and qualitative analysis remains time-consuming and risks overlooking critical trace information, particularly in the case of low-abundance VOCs that define sample-specific characteristics. Untargeted chemometric methods such as hierarchical cluster analysis (HCA) and principal component analysis (PCA), as well as supervised approaches such as partial least-squares-discriminant analysis (PLS-DA), are commonly employed to reduce dimensionality and reveal group patterns in complex chromatographic data sets. However, these techniques often suppress sample-specific features during projection, depend on intensive preprocessing and peak alignment, and may miss chemically important signals that lie outside dominant variance directions or predefined chromatographic regions.^2,9^

In response to these challenges, several analytical strategies have been developed that operate directly on peak-level or image-level representations of GC × GC with HR-TOF-MS data. One widely adopted approach is the peak feature method, which aggregates each detected peak with associated metadata, such as retention time, intensity, and spectral profile, into structured tables for downstream analysis.^10^ Tile-based methods built on this representation commonly employ *Fisher*-ratio (*F*-ratio) analysis to identify discriminative features at low concentration levels.^11–15^ Template matching strategies align peaks across samples using spectral similarity and retention thresholds, often referencing composite chromatograms to define feature regions.^16^ Workflows such as GC Image Investigator support peak alignment and template construction, and can visualize pointwise differences using color-mapped overlays.^7,9^ Digital image (DI) analysis offers a complementary strategy, treating selected chromatographic regions as image segments and applying statistical or machine learning models, such as partial least-squares (PLS) regression or genetic algorithms, for classification or quantification.^3,17^ These methods assume a proportional relationship between pixel intensity and analyte concentration, and have proven effective in classifying botrytized wines and authenticating edible oils.^3,18^ Despite their utility, these approaches face key limitations. Their effectiveness often hinges on analyst-defined parameters such as tile size or segmentation boundaries, particularly in *F*-ratio and DI-based analyses. In complex or untargeted studies, fixed-size regions may capture multiple peaks, partial peaks, or overlapping signals, especially when analyte concentrations vary across samples. Furthermore, such methods rarely adapt to peak-specific *m*/*z* characteristics, limiting their robustness in chemically diverse or highly variable data sets.

Topological Data Analysis (TDA) has emerged as a powerful framework for exploring high-dimensional chemical data sets, offering a model-free approach to capture intrinsic data structure without relying on linear projections or supervised assumptions. In mass spectrometry imaging (MSI),^19^ the Mapper algorithm^20^ is applied to pancreatic tissue data and uncovered spatially organized molecular clusters including islet-associated rings and vascular features, missed by standard methods like Uniform Manifold Approximation and Projection (UMAP).^21^ The persistent homology is employed to extract noise-resistant features from Matrix-Assisted Laser Desorption/Ionization Mass Spectrometry Imaging (MALDI-MSI) for tumor classification.^22^ Earlier, TDA was introduced to the analytical chemistry community,^23^ emphasizing its resilience to noise and relevance for large, complex data sets, while its advantages over PCA was demonstrated for interpreting hyperspectral chemical images.^24^ Mapper has also been applied to laser ablation MS in geochemical studies, where topological networks was used to differentiate spectral signatures from microfossils and mineral matrices.^25^ Despite these promising developments, applications of TDA to comprehensive GC × GC with HR-TOF-MS remain largely unexplored.

In this paper, we introduce a statistically robust and Ball Mapper-based framework for the untargeted analysis of GC × GC with HR-TOF-MS data, addressing key limitations of conventional methods such as PCA, PLS-DA, tile-based *F*-ratio analysis, and Mapper.^20^ Ball Mapper^4^ produces a graph of overlapping balls that captures both local and global geometric structure, without relying on linear projections or class supervision. Unlike PCA, which may obscure chemically meaningful variance,^24^ or PLS-DA, which is vulnerable to overfitting in small-sample, high-dimensional settings,^26^ Ball Mapper captures nonlinear relationships in the full data space. Compared to tile-based *F*-ratio methods,^12,27^ which require careful tuning of segmentation and thresholds, Ball Mapper offers a parameter-efficient alternative that yields interpretable topological summaries. Recent studies in environmental chemistry confirm this promise. Ball Mapper’s advantages over conventional Mapper are demonstrated in pollutant profiling,^28^ and further applied to reveal seasonal contamination patterns in landfill leachate.^29^ Building on this foundation, we propose a statistically robust framework that identifies representative groups of samples by analyzing consistent clustering patterns across multiple views of the data in high-dimensional space.^30^ This approach enhances the interpretability of the analysis and reveals robust structural associations between chemical composition and sample metadata. Although our case study focuses on wines, the method is fully general and applicable to any domains where GC × GC with HR-TOF-MS data are collected.

## 2 Data Acquisition

### 2.1 Chemicals, Samples and Sample Preparation

Benzophenone and sodium chloride (NaCl) were obtained from Microchem, while a mixture of alkanes (C_7_–C_30_), used for calculating linear retention indices, was obtained from Supelco. A total of 34 botrytized wine samples from four countries: Slovakia, Hungary, Austria, and France, were analyzed. Detailed information about these samples is provided in Table 1. In Tokaj winemaking, a putňa (Slovak) or puttonyos (Hungarian) is a unit representing 20–25 kg of botrytized grapes added to 136–140 L of base wine. The number of putňa determines the sweetness, richness, and style of the final wine. Sample preparation involved mixing 6 mL of wine with 0.5 g of NaCl and adding 1 μL of a benzophenone solution in methanol (0.16 mg/L) as an internal standard. All samples were prepared according to the article.^31^ Additionally, the SPME methodology was optimized in a published study.^32^

**Table 1:** List of the Analysed Wine Samples Showing Geographic Origin, Wine Category And Vintage

*k*	**winary**	**wine category**	**country**	**vintage**	**code**
1	Feiler-Artinger	Ruster Ausbruch - Essenz	Austria	2007	AU1
2	Feiler-Artinger	Ruster Ausbruch	Austria	2015	AU2
		- DAC Gelber Muskateller			
3	Feiler-Artinger	Ruster Ausbruch	Austria	2018	AU3
		- DAC Gelber Muskateller			
4	Feiler-Artinger	Ruster Ausbruch	Austria	2017	AU4
		- WB (65%) and WR (35%)			
5	Wenzel	Ruster Ausbruch	Austria	2006	AU5
		- Am Fuße des Berges			
6	Wenzel	Ruster Ausbruch	Austria	2007	AU6
		- Am Fuße des Berges			
7	Wenzel	Ruster Ausbruch - Saz	Austria	2005	AU7
8	Wenzel	Ruster Ausbruch - Saz	Austria	2007	AU8
9	Sajgó Pincészet	Tokaj -3 putňa selection	Hungary	2008	HU1
10	Sajgó Pincészet	Tokaj -5 putňa selection	Hungary	2006	HU2
11	Sajgó Pincészet	Tokaj -6 putňa selection	Hungary	2013	HU3
12	Simkó Pincészet	Tokaj -5 putňa selection	Hungary	2013	HU4
13	Simkó Pincészet	Tokaj -6 putňa selection	Hungary	2014	HU5
14	Simkó Pincészet	Tokaj - essence selection	Hungary	2008	HU6
15	Ostrožovič	Tokaj -3 putňa selection	Slovakia	2002	SK1
16	Ostrožovič	Tokaj -4 putňa selection	Slovakia	2004	SK2
17	Ostrožovič	Tokaj -6 putňa selection	Slovakia	1993	SK3
18	Ostrožovič	Tokaj -6 putňa selection	Slovakia	1999	SK4
19	Ostrožovič	Tokaj -6 putňa selection	Slovakia	2002	SK3
20	Ostrožovič	Tokaj -6 putňa selection	Slovakia	2003	SK6
21	Ostrožovič	Tokaj - essence selection	Slovakia	2000	SK7
22	Tokaj & Co	Tokaj -6 putňa selection	Slovakia	2006	SK8
23	Tokaj & Co	Tokaj - essence selection	Slovakia	1999	SK9
24	Zlatý Strapec	Tokaj -3 putňa selection	Slovakia	1995	SK10
25	Zlatý Strapec	Tokaj -6 putňa selection	Slovakia	1993	SK11
26	Zlatý Strapec	Tokaj -6 putňa selection	Slovakia	2009	SK12
27	Château d’Armajan des Ormes	Sauternes	France	2013	FR1
28	Château Rieussec	Sauternes	France	2011	FR2
29	Château Rieussec	Sauternes	France	2015	FR3
30	Château Coutet	Sauternes	France	2016	FR4
31	Château d’Yquem	Sauternes	France	1995	FR5
32	Château Suduiraut	Sauternes	France	1999	FR6
33	Château Suduiraut	Sauternes	France	2008	FR7
34	Château Suduiraut	Sauternes	France	2017	FR8

### 2.2 Instrumentation

The samples were analyzed using a Pegasus GC × GC with HR-TOF-MS system (LECO Corporation), comprising an Agilent 7890B gas chromatograph (Agilent Technologies) coupled with a HR-TOF-MS (Leco, San Joseph, USA) and a cryogenic modulator. The chromatographic setup included a 30 m × 0.25 mm × 0.25 μm DB-FFAP column (Agilent Technologies) with a highly polar nitroterephthalic-acid-modified polyethylene glycol stationary phase for the first dimension, and a 1 m × 0.25 mm × 0.25 μm Rxi-17Sil column (Restek) with a medium-polar fused silica stationary phase for the second dimension. Helium (99.999% purity) was employed as the carrier gas at a flow rate of 1 mL/min. The oven temperature program started at 40 °C, held for 10 min, then increased at 2 °C/min to 220 °C, where it was maintained for 5 min. Throughout the analysis, the secondary oven operated 5 °C above the primary oven. The modulator was set to 15 °C above the primary oven with an 8*s* modulation period, comprising a 2.40 s hot pulse and a 1.60 s cooling phase. The transfer line temperature was maintained at 250 °C. Mass spectrometry was performed with electron ionization (70 *eV*), an ion source temperature of 250 °C, a detector voltage of 1800 V, a mass range of *m*/*z* 29–600, and a data acquisition rate of 100 spectra/s.

## 3 Method

In this section, we aim to explain statistically robust framework based on Ball Mapper with the symbols listed in Table 2. These notations establish a clear interpretation of mass spectrometry signals and precise description for the procedures of the proposed method. Finally, based on these conventions, we identify representative groups of samples that exhibit similar patterns of mass spectrometry. Figure 1 illustrates the complete analytical workflow, which consists of two main stages: feature extraction and pattern discovery.

**Table 2:** Nomenclature: Sets of Discrete Mass Spectral Vectors for the *k*th Sample in Table 1

symbol	description
*V*_dMS_^*k*^	discrete mass spectral vectors from the discretization of functions in $W^{k}$ (eq 1)
*V*_nMS_^*k*^	normalized discrete mass spectral vectors from *V*_dMS_^*k*^.
*V*_rMS_^*k*^	representative discrete mass spectral vectors:
	subset of *V*_nMS_^*k*^ obtained by Ball Mapper with the spatial resolution ϵ.
*V*_ruMS_^*k*^	relatively unique discrete mass spectral vectors:
	subset of *V*_rMS_^*k*^ at least distance δ from all other elements in *V*_rMS_^*k*′^, *k*′ ≠ *k*.

**Figure 1:**
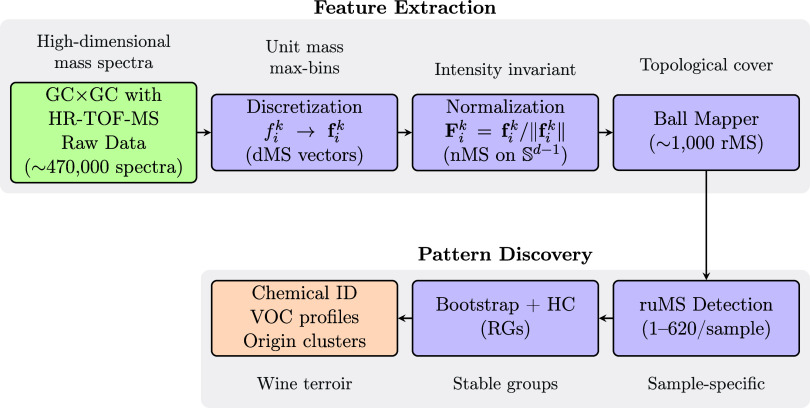
Analytical workflow for topological analysis of GC × GC with HR-TOF-MS data. The two-stage process first extracts representative features through discretization, normalization, and Ball Mapper coverage (top), then identifies sample-specific markers and stable wine groups through statistical validation (bottom). Numbers indicate typical data reduction at each step.

### 3.1 Discretization of HR-TOF-MS Data

The GC × GC coupled with HR-TOF-MS of the *k*th sample is presented by a set of functions:

1 $$\begin{array}{l} W^{k}=\left\{f_{i}^{k}:R^{+}\to R^{+}|i\in I^{k}\right\}\\ k\in K=\left\{1,2,...,K\right\} \end{array}$$

where *k* is the sample number and *K* = 34 is total number of samples in Table 1, a function *f*_*i*_^*k*^ is a high-resolution mass spectrum, called a mass spectral function, and the index set $I^{k}$ indicates all retention times. The index $i\in I^{k}$ presents the first and second retention time, (*t*_*R*1_, *t*_*R*2_) with the lexicographical order of the dependent product space.

We simplify a mass spectral function $f_{i}^{k}:R^{+}\to R^{+}$ by discretizing it into a vector and subsequently projecting this vector onto a high-dimensional unit sphere. This transformation enables robust and interpretable analysis of HR-TOF-MS data, offering the following advantages:

- **Uniformity and Efficiency:** Continuous mass spectral functions are heterogeneous in domains. Discretization into a shared finite-dimensional vector space allows for consistent feature extraction, direct comparison across spectra, and efficient numerical methods.
- **Noise Reduction:** Discretizing over unit-length bins aggregates information in localized windows, reducing sensitivity to noise.
- **Intensity-Invariant Representation:** From the unit length normalization, the direction of the vectors only matters. That is, the projection removes the influence of overall amount in signals, allowing the underlying mass spectrum of chemical compounds to be the primary focus.

This process is formally described as follows. From the domain of *f*_*i*_^*k*^ (dom *f*_*i*_^*k*^), we define two integers *m*_*i*_^*k*^ and *M*_*i*_^*k*^ as the minimum and maximum of the set

$$\left\{n\in Z:[n-\frac{1}{2},n+\frac{1}{2})\;\cap \;\mathrm{dom}f_{i}^{k}\neq ⌀\right\}$$

where Z is a set of integers, then the domain is covered by

2 $$\mathrm{dom}\;f_{i}^{k}\subseteq \overset{M_{i}^{k}}{\underset{n=m_{i}^{k}}{\cup }}\left[n-\frac{1}{2},n+\frac{1}{2}\right)$$

To align all spectra in a common coordinate system, denoting

3 $$m=\min_{k\in K}\left\{\min_{i\in I^{k}}m_{i}^{k}\right\},\;M=\max_{k\in K}\left\{\max_{i\in I^{k}}M_{i}^{k}\right\}$$

we define the *discrete mass spectral* (dMS) vector $f_{i}^{k}\in R^{d}$ associated with the mass spectral function $f_{i}^{k}\in W^{k}$ by

4 $$\begin{array}{l} f_{i}^{k}:\left\{m,...,M\right\}\to R^{+}\;\mathrm{by}\\ f_{i}^{k}\left(n\right)=\{\begin{array}{ll} \max_{x\in \left[n-\frac{1}{2},n+\frac{1}{2}\right]}f_{i}^{k}\left(x\right) & \mathrm{if}\;m_{i}^{k}\leq n\leq M_{i}^{k}\\ 0 & \mathrm{otherwise} \end{array} \end{array}$$

where *d* = *M* – *m* + 1. The collection of such vectors for a sample set (eq 1) is denoted:

5 $$V_{\mathrm{dMS}}^{k}=\left\{f_{i}^{k}\in R^{d}:f_{i}^{k}\left(x\right)\in W^{k},i\in I^{k}\right\}$$

Selecting the maximum value of *f*_*i*_^*k*^(*x*) emphasizes the most prominent features in the mass spectrum, which are chemically relevant.

Now, by normalizing **f**_*i*_^*k*^, the dMS vector is projected onto a high-dimensional sphere $S^{d-1}$ :

6 $$V_{\mathrm{nMS}}^{k}=\left\{F_{i}^{k}=\frac{f_{i}^{k}}{∥f_{i}^{k}{∥}_{l^{2}}}\in S^{d-1}:f_{i}^{k}\in V_{\mathrm{dMS}}^{k},\;i\in I^{k}\right\}$$

where $∥\cdot {∥}_{l^{2}}$ is a discrete $l^{2}$ norm, referring **F**_*i*_^*k*^ as *normalized discrete mass spectral* (nMS) vector. In Figure 2, we illustrate a difference between the original mass spectral function and its nMS vector. The standard GC × GC presents the integration of spectral function $f_{i}^{k}\in W^{k}$ (eq 1) at each retention time $i\in I^{k}$ . The corresponding GC × GC of its nMS vector (eq 6) is the sum of all components in **F**_*i*_^*k*^ ∈ *V*_nMS_^*k*^. The larger quantity of chemical compounds in the standard GC × GC is shown in red. However, the red color in the normalized GC × GC does not mean the quantity of the chemical compound.

**Figure 2:**
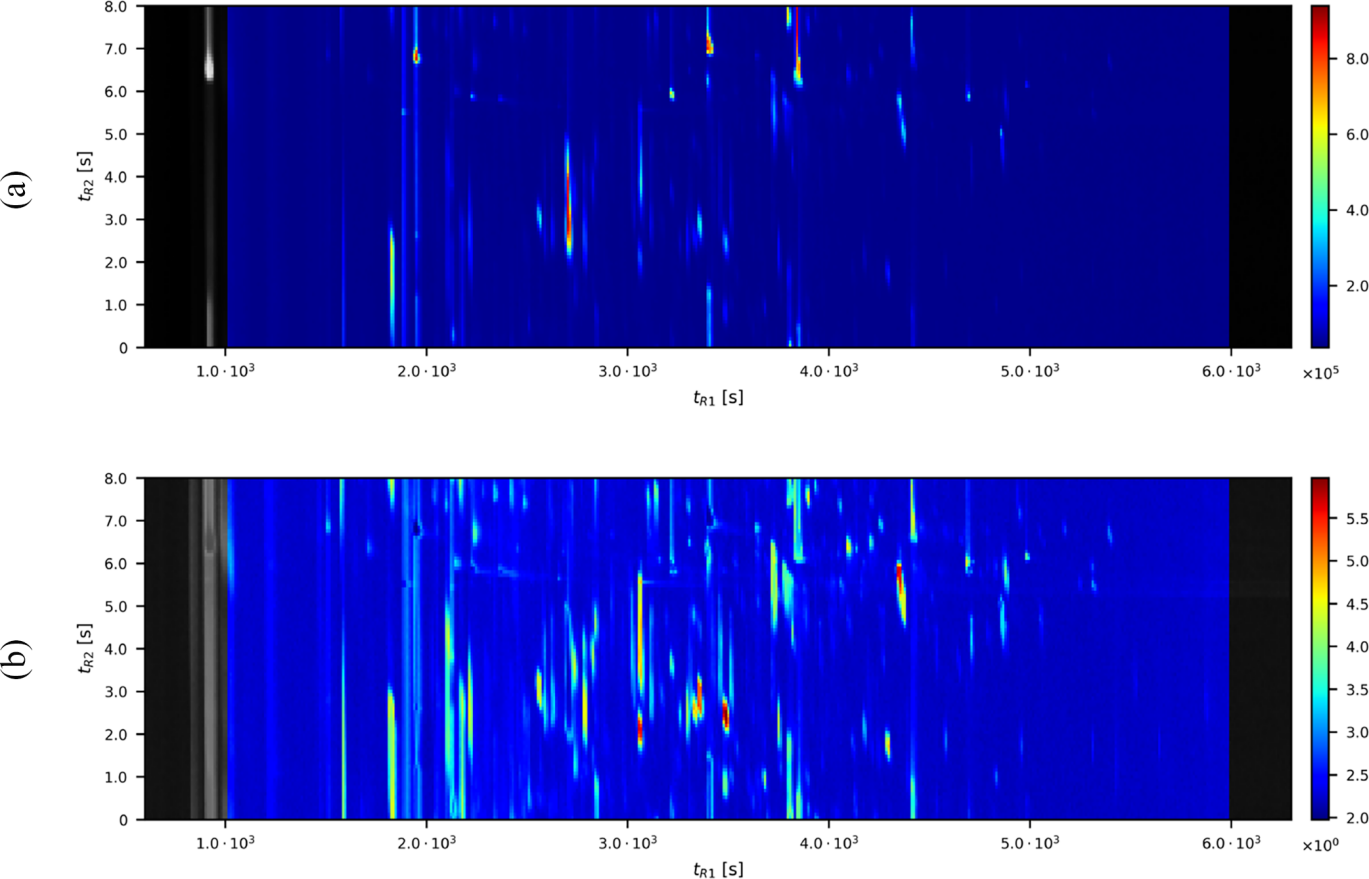
(a) Standard GC × GC of sample *k* = 1 in Table 1 is shown, with color indicating the sum of the dMS vector (eq 4). (b) Normalized GC × GC of (a) is presented, with color indicating the sum of nMS vector (eq 6). Gray regions, corresponding to the beginning and end of the measurement, are excluded from the analysis as they lie outside the region of interest.

**Remark 1.** *We did not apply explicit baseline or blank subtraction prior to vectorization; background signal is mitigated by max-bin discretization* (eq 4), *unit norm projection* (eq 6), *and exclusion of time windows outside the analyzed retention time window* (Figure 2).

### 3.2 Ball Mapper

Ball Mapper^4,5^ is a method from Topological Data Analysis (TDA)^33–35^ designed to facilitate the visualization and exploration of complex, high-dimensional data sets. Let the data set be represented as a finite set of points in a metric space M , equipped with a metric function $m:M\times M\to R^{+}\cup \left\{0\right\}$ . Ball Mapper builds an abstract graph representation of the data by covering M with metric balls of radius ϵ centered at selected *landmarks* in M . Landmarks are selected by the greedy ϵ-net procedure (Algorithm 1 in the Ball Mapper paper^4^): iteratively choose the first uncovered point as a center and mark all points within ϵ as covered until all points are covered. This yields an ϵ-net that (i) covers the data set and (ii) has pairwise landmark distances > ϵ. The resulting Ball Mapper graph consists of:

- **Nodes**: Each node corresponds to the subset of data points contained in a ball of radius ϵ centered at a landmark. Nodes thus represent localized clusters of points in M .
- **Edges**: An edge is drawn between two nodes if at least one data point lies in both. Edges encode continuity between clusters.

By construction, the union of all balls covers the data set, and the scale parameter ϵ directly controls the resolution of the graph. A small ϵ produces many localized nodes, capturing fine-scale structure but possibly leading to overfragmentation. In contrast, a large ϵ yields fewer, broader nodes, simplifying the representation at the cost of masking subtle variations. An illustration of this trade-off is shown in Figure 6.

**Remark 2.** *The choice of the “first” uncovered point depends on the (arbitrary) ordering of the data, the resulting ϵ*-*net (and hence the Ball Mapper graph) is order-dependent rather than uniquely determined by the point cloud; we will later exploit this effective nondeterminism by generating ensembles of ϵ*-*nets from randomized orderings to assess statistical robustness; see* Section 3.5.

### 3.3 Representative Discrete Mass Spectral Vectors

We apply Ball Mapper to *V*_nMS_^*k*^ (eq 6) to identify a small set of representative normalized discrete mass spectral vectors. This set is substantially smaller than the original data set $\left|I^{k}\right|$ , providing a compact and interpretable characterization of *V*_nMS_^*k*^. Crucially, this reduction renders intersample comparison tractable, as the original data contain too many high-dimensional vectors per sample for exhaustive pairwise analysis.

Given the ordered index set $I^{k}$ as defined in eq 6, the data set *V*_nMS_^*k*^ can be regarded as a feature matrix representing the *k*th sample. This matrix has $\left|I^{k}\right|$ rows, where each row is a unit vector in $S^{d-1}$ , corresponding to the ordered elements in *V*_nMS_^*k*^. Specifically, the *i*th row of the feature matrix corresponds to the *i*th element in *V*_nMS_^*k*^. We apply the Ball Mapper algorithm,^4^ implemented in Python,^36^ using the standard Euclidean metric and a fixed ball radius ϵ. In this work, we refer to the landmark points as *representative discrete mass spectral* (rMS) vectors. Formally, the set of rMS vectors is presented by

7 $$V_{\mathrm{rMS}}^{k}=\left\{F_{i}^{k}\in V_{\mathrm{nMS}}^{k}:i\in I_{\mathrm{rMS}}^{k}\subset I^{k}\right\}$$

where $I_{\mathrm{rMS}}^{k}$ is the index set of landmarks. These vectors are chosen to satisfy the following two properties:

8 $$V_{\mathrm{nMS}}^{k}\subset \underset{i\in I_{\mathrm{rMS}}^{k}}{\cup }B_{\epsilon }\left(F_{i}^{k}\right)$$

9 $$\left|F_{i_{1}}^{k}-F_{i_{2}}^{k}\right|>\epsilon ,\forall i_{1},i_{2}\in I_{\mathrm{rMS}}^{k}\;\mathrm{and}\;i_{1}\neq i_{2}$$

where $B_{\epsilon }\left(F_{i}^{k}\right)=\left\{F\in R^{d}\left|\right|F-F_{i}^{k}|<\epsilon \right\}$ denotes an open Euclidean ball of radius ϵ centered at the landmark **F**_*i*_^*k*^. Each node in the Ball Mapper graph corresponds to the set of data points within a given ball:

10 $$N_{\epsilon }\left(F_{i}^{k}\right)=V_{\mathrm{nMS}}^{k}\cap B_{\epsilon }\left(F_{i}^{k}\right),i\in I_{\mathrm{rMS}}^{k}$$

This construction satisfies the relation, $V_{\mathrm{nMS}}^{k}\subset \cup_{i\in I_{\mathrm{rMS}}^{k}}N_{\epsilon }\left(F_{i}^{k}\right)$ , indicating that the node sets *N*_ϵ_(**F**_*i*_^*k*^) form a cover of *V*_nMS_^*k*^. Thus, each node can be interpreted as a local cluster centered at an rMS vector.

### 3.4 Identification of Relatively Unique Discrete Mass Spectral Vector

We next identify *relatively unique discrete mass spectral* (ruMS) vectors within each sample. A ruMS vector is defined as a normalized discrete mass spectral vector in *V*_nMS_^*k*^ that lies at least a certain distance from every nMS vector of another sample $W^{k\prime }$ with *k*′ ≠ *k*. The qualifier “relatively” indicates that uniqueness is assessed against other samples in the data set rather than the full chemical feature space. Since comparing all nMS vectors across samples is computationally expensive, we instead compare only the representative set *V*_rMS_^*k*^ ⊂ *V*_nMS_^*k*^ obtained via Ball Mapper, thereby reducing complexity while preserving key structural information.

For each sample index k∈K , we define a subset of indices $I_{\mathrm{ruMS}}^{k}\subset I_{\mathrm{rMS}}^{k}$ such that the corresponding vectors in *V*_rMS_^*k*^ are well-separated from rMS vectors in all other samples:

11 $$I_{\mathrm{ruMS}}^{k}=\left\{i\in I_{\mathrm{rMS}}^{k}:\left|F-F_{i}^{k}\right|>3\delta ,\forall F\in \underset{k^{\prime }\neq k}{\cup }V_{\mathrm{rMS}}^{k\prime }\right\}$$

where 3δ is to ensure nearby nMS vectors of an ruMS vector stay at least δ away from other samples. This yields the ruMS vectors for the *k* ^th^ sample:

12 $$V_{\mathrm{ruMS}}^{k}=\left\{F_{i}^{k}\in V_{\mathrm{rMS}}^{k}:i\in I_{\mathrm{ruMS}}^{k}\right\}$$

By proposed construction, the inclusion *V*_ruMS_^*k*^ ⊂ *V*_rMS_^*k*^ ⊂ *V*_nMS_^*k*^ holds, implying the cardinality relation $\left|I_{\mathrm{ruMS}}^{k}\right|\leq \left|I_{\mathrm{rMS}}^{k}\right|\leq \left|I^{k}\right|$ . Furthermore, each **F**_*i*_^*k*^ ∈ *V*_ruMS_^*k*^ satisfies the following locality condition:

13 $$\mathrm{F̅}\in B_{\delta }\left(F_{i}^{k}\right)\cap V_{\mathrm{nMS}}^{k}\Rightarrow \left|F-\mathrm{F̅}\right|>\delta ,\forall F\in \underset{k^{\prime }\neq k}{\cup }V_{\mathrm{nMS}}^{k\prime }$$

Each ruMS vector is not only separated from the rMS vectors of other samples but also preserves this separation for all neighboring nMS vectors within distance δ. Thus, ruMS vectors identify locally isolated regions that capture sample-specific spectral uniqueness relative to other samples.

### 3.5 Representative Groups of Samples

The analysis presented in the previous sections focuses on identifying ruMS vectors that characterize each sample, emphasizing local chemical distinctiveness. In this section, we aim to identify *representative groups* (RGs) of samples that exhibit stable patterns of similarity. To achieve this goal, we propose a clustering analysis based on the distribution of ruMS vectors with the Ball Mapper across randomized views of the data in the high-dimensional space. Let us enumerate ruMS vectors from all samples:

14 $$V_{\mathrm{ruMS}}=\underset{k\in K}{\cup }V_{\mathrm{ruMS}}^{k}=\left\{v_{j}\in S^{d-1}:j\in J\right\}$$

where J={1,...,J} and $J=\sum_{k\in K}\left|I_{\mathrm{ruMS}}^{k}\right|$ .

To explore the structure of *V*_ruMS_ in multiple views, we randomize Ball Mapper view s∈S={1,2,...,S} , *S* = 1,000 and compute the frequency matrix

15 $$M^{\left(s\right)}\in {\left[0,1\right]}^{K\times L^{\left(s\right)}}$$

where *L* ^(*s*)^ is the number of nodes and can be changed for each shuffle s∈S , and apply hierarchical clustering to its *K* rows while varying Q∈Q={2,...,15} . To assess stability, we bootstrap the rows of **M** ^(*s*)^ and, for each (s,Q)∈S×Q , compute the Adjusted Rand Index ARI­(*s*,*Q*)^37^ between the clusters of the original matrix **M** ^(*s*)^ and those obtained from 1,000 bootstrap resamples. We then define the consistency score of clustersthe consistency score of clustering, which quantifies its statistical robustness and reliability:

16 P(Q)=|{s∈S:ARI(s,Q)≥0.5}|

and determine the optimal number of clusters as

17 $$Q^{*}=\arg\max_{Q\in Q}P\left(Q\right)$$

In our study, the cosine dissimilarity is used for HC, and the procedure in eq 17 yields that the maximum consistency of the clusters is obtained by the Q* = 11.

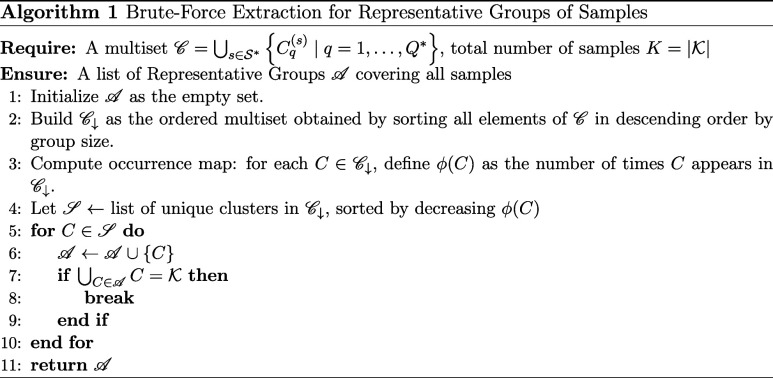



Each clustering result yields a partition of the sample index set K into Q* groups. Denoting $S^{*}=\{s\in S:$ $\mathrm{ARI}\left(s,Q^{*}\right)\geq 0.5\}$ , we observe that although the cluster boundaries in ${\left\{M^{\left(s\right)}\right\}}_{s\in S*}$ vary slightly across shuffles, many stable groups of samples emerge consistently. Let us denote the multiset of all clusters as

18 $$C=\underset{s\in S^{*}}{\cup }C^{\left(s\right)},C^{\left(s\right)}=\left\{C_{q}^{\left(s\right)}:q=1,2,...,Q^{*}\right\}$$

where each $C_{q}^{\left(s\right)}\subset K$ represents a cluster of samples in the *s* ^th^ shuffle satisfying the following:

$$\overset{Q^{*}}{\underset{q=1}{\cup }}C_{q}^{\left(s\right)}=K,\;\mathrm{and}\;C_{q_{1}}^{\left(s\right)}\cup C_{q_{2}}^{\left(s\right)}=⌀,\mathrm{if}\;q_{1}\neq q_{2}$$ 19

Algorithm 1 aims to extract groups of samples that recur most frequently in the multiset C , using a brute-force strategy to construct RGs of samples.

## 4 Results and Discussion

### 4.1 Ball Mapper-Derived Spectral Summaries and Clusters

To aid interpretation of the proposed method and its results, Table 2 shows the notation for different sets of discrete mass spectral vectors, and Table 3 reports the numbers of representative (rMS) and relatively unique (ruMS) vectors per sample. The rMS vectors *V*_rMS_^*k*^ are obtained by applying Ball Mapper with resolution ϵ = 0.08 to the normalized mass spectral vectors *V*_nMS_^*k*^, reducing the original data set (about 470,000 vectors per sample) to a sparse yet informative set of localized summaries.

**Table 3:** Code is from Table 1 ^a^

*k*	code	|*V*_rMS_^*k*^|	|*V*_ruMS_^*k*^|	*k*	code	|*V*_rMS_^*k*^|	|*V*_ruMS_^*k*^|
1	AU1	964	65	18	HU2	1417	125
2	AU2	1757	620	19	HU3	1272	133
3	AU3	828	105	20	HU4	1205	61
4	AU4	613	14	21	HU5	1252	42
5	AU5	743	13	22	HU6	1339	78
6	AU6	682	7	23	SK1	1144	34
7	AU7	1087	19	24	SK2	1151	73
8	AU8	1081	34	25	SK3	1075	30
9	FR1	981	10	26	SK4	1100	64
10	FR2	928	19	27	SK5	840	9
11	FR3	715	1	28	SK6	998	11
12	FR4	1419	142	29	SK7	1168	62
13	FR5	1308	170	30	SK8	1375	219
14	FR6	1149	101	31	SK9	1300	291
15	FR7	1040	22	32	SK10	1256	61
16	FR8	836	10	33	SK11	1331	82
17	HU1	977	29	34	SK12	978	36

a The corresponding numbers of rMS (eq 7) and ruMS (eq 12) vectors are listed below.

The ruMS vectors *V*_ruMS_^*k*^ ⊂ *V*_rMS_^*k*^ are selected by a geometric criterion: they must lie at least a distance 3δ = 0.072 away from any rMS vectors in all other samples. This ensures that each ruMS vector represents a sample-specific region in high-dimensional space that is both locally supported (by neighboring nMS vectors within distance δ) and relatively unique (well-separated from all other samples). Variation in ruMS counts across samples (e.g., AU2 with 620 vs FR3 with 1) reflects differences in chemical uniqueness and diversity among the wines.

Figure 3 visualizes the structure of all ruMS vectors, $V_{\mathrm{ruMS}}=\cup_{k\in K}V_{\mathrm{ruMS}}^{k}$ , using Ball Mapper with a coarser resolution γ = 3ϵ. Each node represents a group of geometrically close ruMS vectors, and node colors encode the proportion of vectors from each country. For example, in the top-left panel, red nodes contain only Austrian ruMS vectors, while lighter shades indicate mixtures with other countries. This visualizition highlights the degree of chemical separability across geographic origins: country-specific nodes mark distinct profiles, whereas mixed nodes and overlaps indicate shared or less distinctive features. The resulting graph provides a topological summary of chemically informative differences, demonstrating that ruMS vectors serve as effective discriminative markers of wine origin. Together with Table 3, this figure illustrates the proposed framework’s ability to condense complex HR-TOF-MS data into interpretable maps of wine-specific chemical diversity.

**Figure 3:**
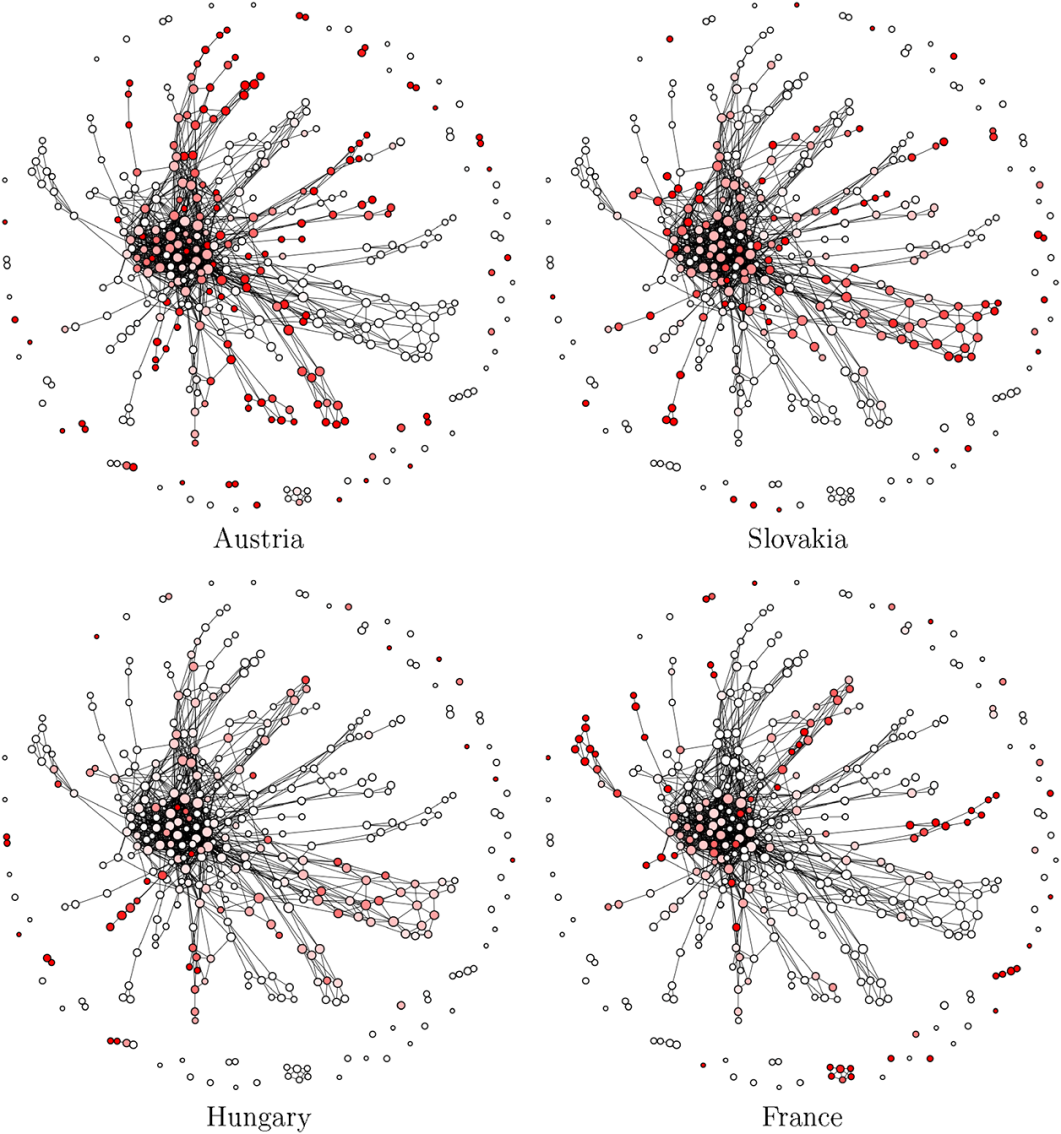
Four Ball Mapper graphs are shown with node colors indicating the proportion of *V*_ruMS_^*k*^ originating from that corresponding country. Nodes are fully red if they contain only *V*_ruMS_^*k*^ from that country, white if they contain none, and shaded accordingly in between.

While Figure 3 provides a visual overview of ruMS distributions across samples, it does not directly summarize similarities or differences between individual wines. To address this, we identify *representative groups* (RGs) of wines using Algorithm 1, which detects statistically identified clusters of samples that frequently co-occur within localized regions of the high-dimensional spectral space. These regions correspond to nodes in the Ball Mapper graph, and co-occurrence frequencies are quantified by the frequency matrix in eq 15.

Table 4 shows the selection of the optimal cluster number Q* by maximizing the consistency score *P*(*Q*) in eq 16. As given in eq 17, the maximum occurs at Q* = 11, indicating that 11 clusters provide the most stable and reproducible partition under hierarchical clustering of the frequency matrix (eq 15). Table 4 also shows that the highest consistency arises at an intermediate cluster count, not at the extremes of very few or many clusters.

**Table 4:** Consistency Score *P*(*Q*) is the Number of Shuffles with ARI­(*s*, *Q*) ≥ 0.5 for Each Q∈Q ; See eq 16 ^a^

*Q*	2	3	4	5	6	7	8	9	10	**11**	12	13	14	15
*P*(*Q*)	34	795	871	924	971	980	981	976	986	**993**	991	970	928	755

a The maximum value is attained at *Q* = 11.

Using the result Q* = 11 from Table 4, we present RGs of wines from Algorithm 1 in Table 5. Each row contains the indices of wine samples assigned to a representative group, and the values ϕ­(*C*) and *A*(*C*). The occurrence frequency ϕ­(*C*) shows repeated observations of the cluster *C* in the multiset (eq 18) according to the Algorithm 1. The reproducible consistency *A*(*C*) is computed by

20 $$A\left(C\right)=\frac{1}{\left|S\left(C\right)\right|}\sum_{s\in S\left(C\right)}\mathrm{ARI}\left(s,Q^{*}\right),S\left(C\right)=\left\{s\in S^{*}:C\in C^{\left(s\right)}\right\}$$

where $C^{\left(s\right)}$ is defined in eq 18. It explains how consistently the cluster *C* can be reproducible whenever it is observed. Table 5 numerically confirms the following facts:

- Small groups contain few samples but maintain highly stable identity across random shuffles, indicating strong local chemical similarity.
- Larger groups encompass more diverse samples, possibly reflecting generalized chemical profiles.
- Some samples appear in multiple groups, reflecting overlapping high-dimensional relationships not captured by exclusive clusters.

**Table 5:** We Present the Representative Groups (RGs) of Wines Obtained by Algorithm 1^a^

RG	C⊂K={1,2,...,K(=34)}	ϕ(*C*)	*A*(*C*)
1	{29} = {FR3}	993	0.588
2	{6} = {AU6}	993	0.588
3	{4} = {AU4}	987	0.587
4	{7, 8} = {AU7, AU8}	986	0.588
5	{10, 11, 18, 22, 23, 24, 25}	745	0.591
	= {HU2, HU3, SK4, SK8, SK9, SK10, SK11}		
6	{1, 5} = {AU1, AU5}	745	0.591
7	{28, 33} = {FR2, FR7}	635	0.590
8	{20, 21, 26} = {SK6, SK7, SK12}	420	0.591
9	{34} = {FR8}	293	0.585
10	{12, 13} = {HU4, HU5}	169	0.595
11	{9, 19} = {HU1, SK5}	167	0.593
12	{1} = {AU1}	166	0.576
13	{33} = {FR7}	164	0.572
14	{16, 20, 21, 26} = {SK2, SK6, SK7, SK12}	157	0.590
15	{10, 11, 12, 18, 22, 23, 24, 25}	149	0.584
	= {HU2, HU3, HU4, SK4, SK8, SK9, SK10, SK11}		
16	{28, 31, 32, 33} = {FR2, FR5, FR6, FR7}	136	0.590
17	{5, 28} = {AU5, FR2}	135	0.574
18	{20, 34} = {SK6, FR8}	118	0.580
19	{16, 26} = {SK2, SK12}	90	0.577
20	{5} = {AU5}	88	0.579
21	{10, 11, 18, 22, 24, 25}	87	0.568
	= {HU2, HU3, SK4, SK8, SK10, SK11}		
22	{20, 21} = {SK6, SK7}	67	0.577
23	{2, 3, 9, 14, 15, 16, 17, 27, 30}	56	0.618
	= {AU2, AU3, HU1, HU6, SK1, SK2, SK3, FR1, FR4}		

a The second column shows which wines are grouped together. The value *ϕ*(*C*) is the number of repeated observations of the cluster *C* in all clusters (eq 18) according to the Algorithm 1. The last column (eq 20) evaluates the reproducible consistency of the cluster *C* under random shuffles.

Finally, Figures 4 and 5 are a graphical presentation of RGs in Table 5. In comparison to Figures 3 and 4 clearly shows which wines are grouped together based on similarities in their ruMS vectors and which wines are separated. A detailed interpretation is provided in the following section.

**Figure 4:**
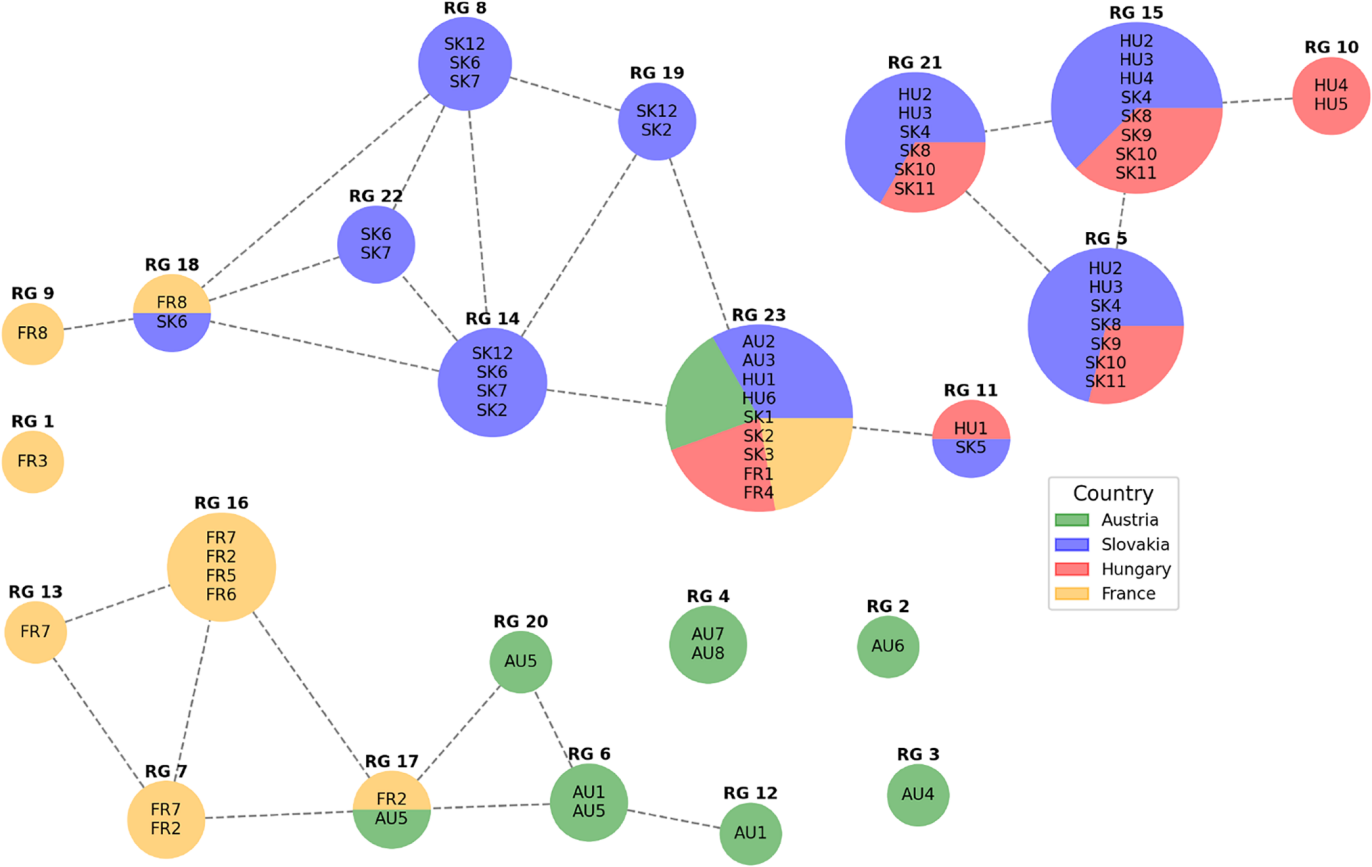
Representation groups (RGs) of wines and their similarities are visualized by a graph with a pie chart colored by country. The size of each pie chart is proportional to the number of wines in the corresponding RG, and dotted lines between pie charts indicate shared wine samples between RGs.

**Figure 5:**
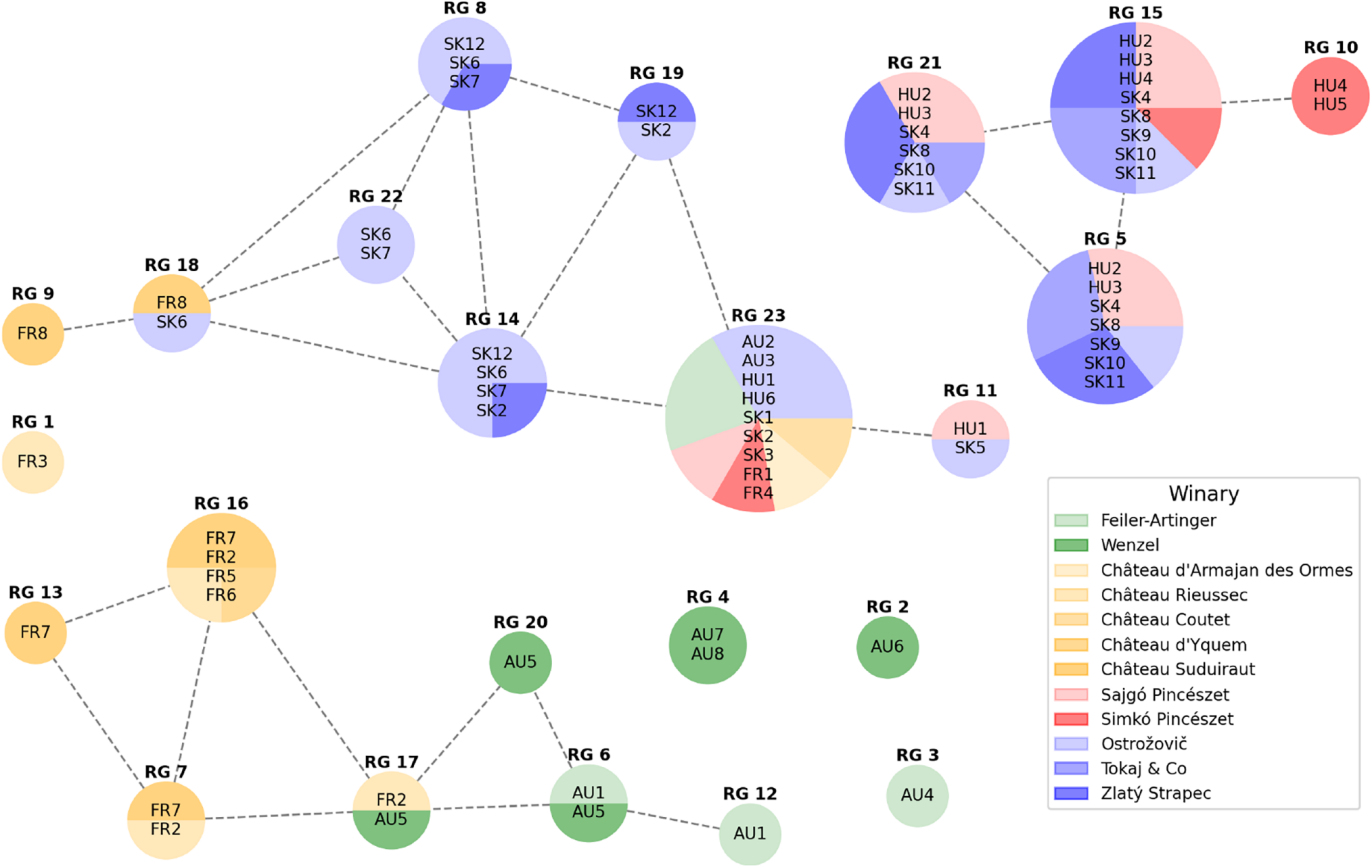
Representation groups (RGs) of wines and their similarities are visualized by a graph with a pie chart colored by winary.

### 4.2 Topological Chemometrics of Wine Spectra

#### 4.2.1 Parameters Settings

To construct rMS vectors via Ball Mapper, we set the resolution to ϵ = 0.08. This choice is supported by replicate measurements of wine sample FR6 (Table 1), where over 95% of pairwise distances fall below this threshold, indicating reproducibility within ϵ and potential chemical differences beyond it.

For ruMS detection, we define the minimum separation δ = *rϵ* with 3δ < ϵ < 4δ, ensuring local support from within-sample neighbors (within δ) and separation from other samples (beyond 3δ). With *r* = 0.3, we obtain δ = 0.024, satisfying 0.072 < ϵ < 0.096 and providing a robust buffer to isolate chemically distinctive regions. For visualization across samples, we apply Ball Mapper at a coarser resolution γ = 3ϵ = 0.24, which merges similar features while avoiding overfragmentation, yielding stable and interpretable wine clusters.

#### 4.2.2 The Effect of Spatial Resolution

In Figure 6, we present the normalized GC × GC, *V*_nMS_^17^ in Table 1 along with the corresponding the Ball Mapper of varying spatial resolution ϵ. The color of each node *N*_ϵ_(**F**_*i*_^17^), $i\in I_{\mathrm{rMS}}^{k}$ , is determined by the average of the total intensities of the vectors contained in the node:

21 $$\left\{\sum_{n=1}^{d}F_{n}|F\in N_{\epsilon }\left(F_{i}^{17}\right)\subset S^{d-1}\right\}$$

where *F*_*n*_ denotes the *n* ^th^ component of the vector $F\in S^{d-1}$ . The color range used in the normalized GC × GC is consistent with that of the Ball Mapper visualizations. As seen from the nodes rendered in warmer colors (corresponding to higher total intensities), increasing ϵ leads to a coarser representation that simplifies the structure of the resulting graph. In this study, we fix the radius parameter to ϵ = 0.08 for all wines, with the choice explained in Section 4.2.1.

**Figure 6:**
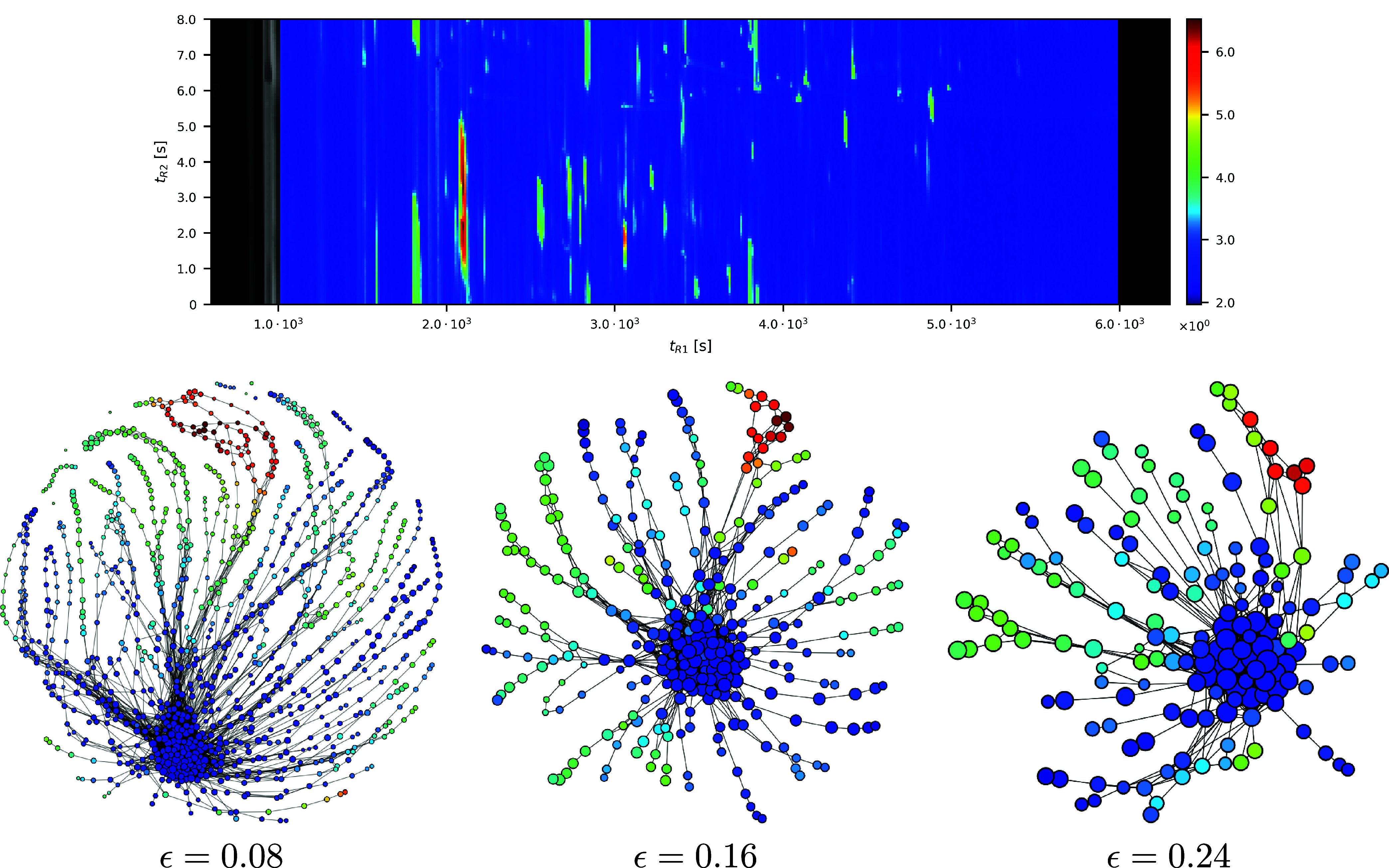
Top panel shows the normalized GC × GC chromatogram for wine *V*_nMS_^17^ in Table 1. The bottom panels display three Ball Mapper graphs of the same sample, constructed using progressively larger spatial resolutions.

#### 4.2.3 Qualitative Comparison of PCA, UMAP, tSNE, and Ball Mapper

A common strategy to reduce the computational cost of high-dimensional mass spectral data is to apply dimensionality reduction methods such as Principal Component Analysis (PCA), Uniform Manifold Approximation and Projection (UMAP),^21^ or t-distributed Stochastic Neighbor Embedding (tSNE).^38^ These project $V_{\mathrm{nMS}}^{k}\subset R^{d}$ into a lower-dimensional space $R^{\mathrm{d̃}}$ with *d̃* ≪ *d*, aiding visualization and downstream clusters but often distorting geometric relations in the original space. By contrast, Ball Mapper operates directly on *V*_nMS_^*k*^, identifying a compact yet structurally informative representation without dimensional compromise.

**Remark 3.** *Ball Mapper does not reduce dimension like PCA, UMAP, or t-SNE, but simplifies data by summarizing its structure as a graph that highlights clusters, continuous trends, and their relations. Its primary role is topological summarization and visualization rather than dimensionality reduction*.

Figure 7 illustrates these differences in *V*_nMS_^1^. PCA, UMAP, and t-SNE reduce to *d̃* = 2, while Ball Mapper is shown at resolution ϵ = 0.2 (chosen for clarity). To enable comparison, node colors correspond to normalized GC × GC intensities in Figure 2b. In PCA, many red- and blue-shaded points overlap, indicating limited separation. UMAP yields curved, intertwined clusters, while t-SNE suggests possible clustering but requires additional clustering step for meaningful summarization. In contrast, Ball Mapper (bottom-right) reveals clusters of similar nMS vectors and their interconnections without dimensionality reduction, offering improved separation and interpretability of distances.

**Figure 7:**
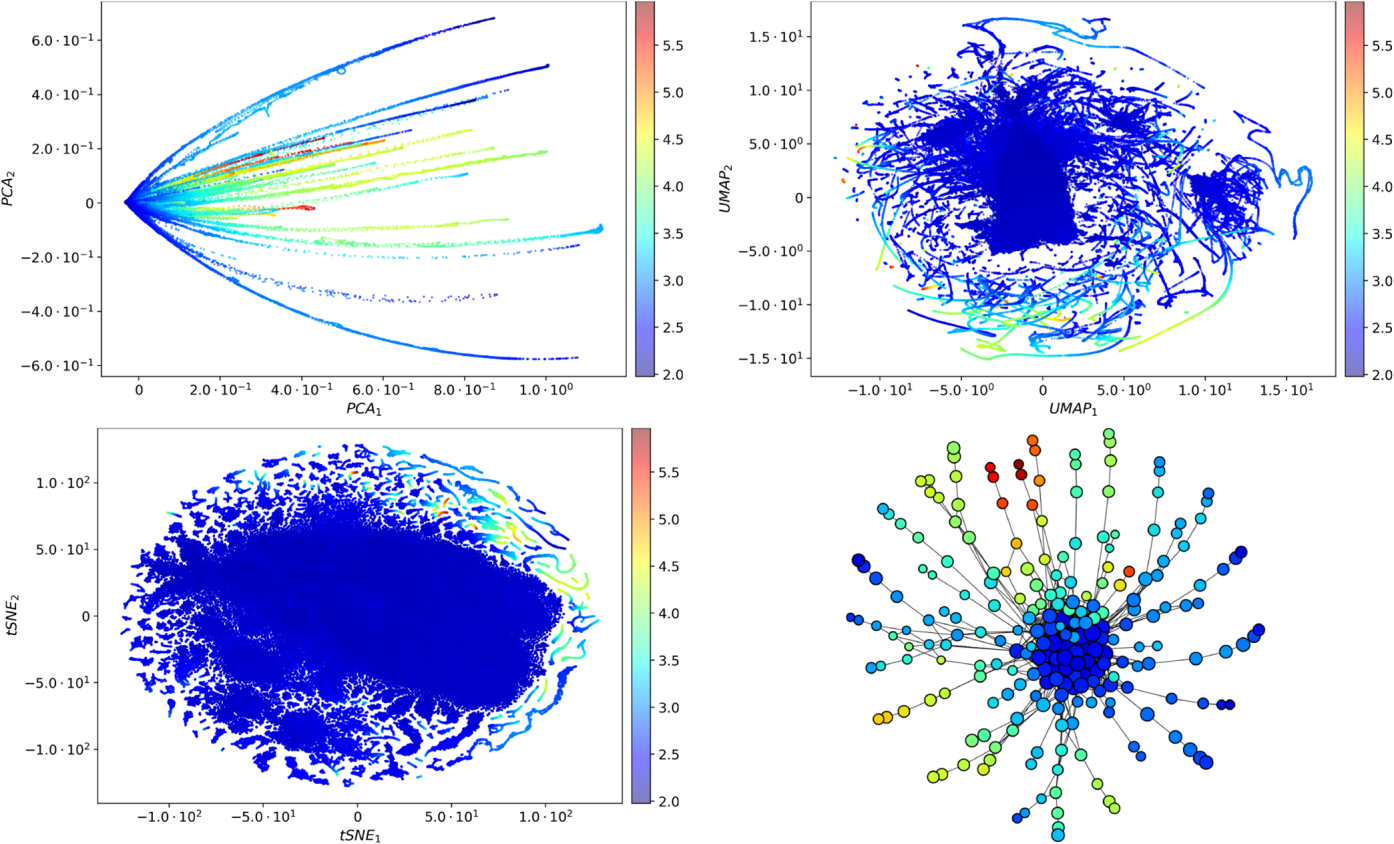
PCA, UMAP, tSNE, and Ball Mapper of wine *k* = 1 (Table 1) for *V*_nMS_^1^ (eq 6): The Ball Mapper graph is shown at ϵ = 0.2 for legibility; all analyses elsewhere use ϵ = 0.08; see also Figure 6 for resolution effects.

#### 4.2.4 Ball Mapper Analysis Reveals Chemically Distinct Wine Clusters and Markers

The Ball Mapper algorithm provided a powerful topological framework to distill the complex GC × GC with HR-TOF-MS data into chemically interpretable patterns. With this method, 2,792 (= |*V*_ruMS_|) distinct discrete mass spectral features (*m*/*z* distributions) across the 34 botrytized wine samples were extracted; see the Supporting Information Table_1S.xlxs. From these, 1191 features were matched to compounds or compound classes. This set includes 334 identified compounds assigned to known volatile organic compound (VOC) classes (e.g., esters, furans, alcohols, terpenes, lactones) and an additional 46 distinct features that could only be tentatively classified by VOC class; see the Supporting Information Table_2S.xlxs. These ruMS vectors serve as sample-specific chemical fingerprints. Some ruMS vectors correspond to known flavor constituents of botrytized wines, and their distribution reflects the full chemical complexity of the samples.

Table 3 highlights the number of rMS and ruMS vectors identified per wine. Chemical uniqueness varied across samples: Austrian Ausbruch AU2 exhibited 620 ruMS features, while French Sauternes FR3 had only one. The observed disparities suggest that some wines contain rich diversity of unique compounds, whereas others share most of their VOC with the broader set. Tokaj wines (Slovak and Hungarian) showed consistently high ruMS counts (e.g., SK8 with 219; SK9 with 291), indicating diverse VOC profiles. By contrast, certain Sauternes (FR3, FR8), Slovak Tokaj (SK5, SK6) and Austrian wines (AU4, AU6, AU5) showed few unique features, implying greater overlap or dominance of common botrytized wine compounds. This is chemically meaningful, as noble rot produces shared aromas, yet specific ruMS vectors still distinguish origin. The wide range of ruMS counts underscores that this approach is sensitive to subtle compositional differences. Even a single unique marker, as observed in FR3, can be significant, while the presence of hundreds of ruMS suggests a sample with distinct chemical complexity.

Critically, the ruMS features are not abstract statistical constructs but actual discrete mass spectral signatures traced back to chemical identities. Furfuryl ethyl ether was uniquely prominent in Hungarian Tokaj wines, likely formed from furfuryl alcohol during oak barrel fermentation and aging,^39,40^ reflecting regional barrel-aging practices. Creosol, a volatile phenolic from lignin degradation during barrel toasting, was consistently detected in both Slovak and Hungarian Tokaj wines, highlighting traditional oak maturation.^41,42^ Slovak Tokaj wines also showed phenolic- and ester-rich profiles, with unique ruMS compounds such as E-3-hexenoic acid and diethyl 2-hydroxy-pentanedioate, contributing sweet, spicy, and fermentative notes.^43–46^ In contrast, French Sauternes featured ruMS markers like 3-(methylthio)-1-propanol and dihydroxyacetone, linked to Botrytis metabolism,^47,48^ along with benzoic acid derivatives and γ-butyrolactone, enhancing honeyed and creamy aromas.^3,49–51^ Sauternes displayed higher alcohols, aldehydes, and ketones, whereas Tokaj wines were defined by phenolics and ester-driven sweetness. Notably, one Slovak sample (SK5) contained only benzyl methyl ketone as a characteristic ruMS, while a Sauternes (FR3) lacked clear unique markers. These cases illustrate that some wines exhibit such high chemical similarity that even sensitive analysis yields few distinguishing features, a challenge for authentication studies where classification depends on identifying unique spectral signatures. This represents a relevant insight for authentication studies, as such wines may be more difficult to classify using conventional methods.

The Ball Mapper representation group (RG) analysis in Figures 4 and 5 provides a higher-level view of how chemical markers cluster the wines. Using 1000 observations, statistically robust RGs were identified. Each RG is shown as a pie-chart node, with size reflecting the number of wines and slice colors indicating composition by wine country or producer. Dotted lines indicate overlaps, highlighting shared VOC profiles across clusters. Grouping by ruMS vectors strongly aligns with winemaking practices and geographic origin. Tokaj-region wines (Slovak and Hungarian) largely cluster together. A dominant Tokaj cluster, observed 745 times, combined seven wines (HU2, HU3, SK4, SK8, SK9, SK10, SK11) and featured classic botrytized VOCs such as benzaldehyde, phenylethyl alcohol, diverse ethyl esters (e.g., ethyl hexadienoate, ethyl nonanoate), and 4-ethylphenol. Another Tokaj cluster (RG14), observed 157 times, connected Slovak samples (SK2, SK6, SK7, SK12) was marked by cis-5-butyldihydro-4-methyl-2­(3H)-furanone and benzeneacetaldehyde, reflecting the sweet lactones and phenolic aldehydes typical of the region.

The proposed analysis also highlighted individual wines with highly unique profiles. Notably, two Austrian Ausbruch samples (AU6 and AU4) consistently formed isolated clusters in nearly every iteration, appearing as singleton RGs in 993 and 987 cases, respectively. This shows that each wine possesses a distinctive volatile profile, likely reflecting unique grape varietals or winemaking practices in Rust. Both AU4 and AU6 also had among the highest ruMS counts in Table 3, supporting their chemically singular status. Similarly, certain Sauternes wines stood apart. Sample FR3, though chemically less complex in ruMS number, appeared independently in 993 shuffles, while FR7 and FR8 each formed distinct clusters in many iterations. These results highlight that even within a single appellation (Sauternes), significant bottle-to-bottle differences occur, likely driven by vineyard-specific factors or vintage variation, and can be revealed by this untargeted approach.

Importantly, the proposed framework also revealed cross-regional commonality in volatile profiles. A recurring cluster (56 times) grouped wines from Austria (AU2, AU3), Hungary (HU1, HU6), Slovakia (SK1, SK2, SK3), and France (FR1, FR4). These wines shared prevalent fermentation-derived compounds, including ethyl 9-decenoate, ethyl decanoate, phenylethyl alcohol, benzaldehyde, diethyl succinate, and isoamyl octanoate (3-methylbutyl octanoate). The existence of such a mixed-origin cluster highlights ubiquitous aroma fingerprints in sweet wines (e.g., fruity esters, succinate derivatives), which can cause overlaps between, for example, Tokaj and Sauternes. However, most Ball Mapper nodes were dominated by single-origin wines, indicating strong separability by region. In the Ball Mapper graph of ruMS vectors (Figure 3), many nodes were country-specific (e.g., red nodes for Austrian ruMS), implying that Austrian, Tokaj, and French wines occupy distinct chemical regions with limited overlap. Mixed-color nodes, corresponding to shared volatiles, were rare. Together with the RG analysis, this topological separation confirms that Ball Mapper features capture genuine chemical differences aligned with geography and producer practices. Slovak and Hungarian Tokaj samples clustered together, Austrian Rust Ausbruch wines grouped distinctly, and French Sauternes formed separate clusters, as shown in Figures 4 and 5. The consistency between wine origin (region/producer) and the Ball Mapper groupings lends credence to reliability of this approach for classification and authenticity purposes. In essence, the proposed algorithm transformed thousands of peaks into a a powerful topological framework where both global patterns (e.g., Sauternes vs Tokaj vs Ausbruch) and local idiosyncrasies emerge naturally from the data.

#### 4.2.5 Comparison with Conventional Chemometric Methods

These results demonstrate that the TDA-based approach can augment or outperform traditional chemometric methods in analyzing complex chromatographic–mass spectrometric data. Conventional multivariate methods like principal component analysis (PCA) and partial least-squares-discriminant analysis (PLS-DA) are often applied to data sets of GC × GC with HR-TOF-MS, but they have well-documented limitations in untargeted workflows. For example, PCA projects data into a low-dimensional space optimized for capturing variance, but this can obscure chemically relevant variation related to minor or sample-specific constituents. Subtle yet important VOC signals (e.g., a trace oak-derived phenol unique to one region) may lie outside the dominant variance directions and thus get suppressed in a PCA score plot.^2,9^ Similarly, supervised methods like PLS-DA risk overfitting when the number of variables vastly exceeds the number of samples (the “small n, large p” problem common in metabolomics).^26^ Indeed, PLS-DA can find separations that reflect noise rather than true chemistry if not rigorously validated. In our case, over 470,000 mass spectra per sample reduced only to thousands, a naive PLS-DA risks overfitting by capturing chance correlations. By contrast, the Ball Mapper algorithm makes no a priori assumptions of linearity or class labels, and it considers the full nonlinear relationships in the native high-dimensional space data. Thus, even low-intensity features or those occurring in a limited number of samples can be retained by Ball Mapper, either as distinct nodes or as part of unique clusters, ensuring that chemically significant minority variance is preserved.

Another challenge with classical approaches is the extensive preprocessing required for chromatographic data. Peak-based analysis demands peak picking, retention time alignment, background correction, and normalization before multivariate analysis. Misalignment or misidentification of peaks can directly distort PCA or PLS-DA outcomes. By contrast, the proposed method bypassed strict alignment by clustering similar mass spectra and using the Ball Mapper cover to organize features, tolerating minor retention time shifts as long as spectral signatures remain consistent. This provides robustness against chromatographic variability: Ball Mapper groups overlapping peaks or shifted signals based on spectral similarity in high-dimensional space. Thus, signals of the same compound across samples cluster together even with retention time differences of several seconds, unlike rigid binning or alignment approaches.

Tile-based Fisher ratio (*F*-ratio) and digital image (DI) segmentation have been applied to GC × GC data, including botrytized wines.^3,31^ These approaches identify discriminatory regions based on chromatographic pixel intensities or predefined areas, but depend heavily on analyst-defined parameters such as tile size, boundaries, and significance thresholds. Inappropriate settings can merge distinct compounds, split peaks, or capture only partial signals. Moreover, they assume pixel intensity reflects concentration and often neglect full spectral information (*m*/*z*), making them insensitive to unique low-intensity markers with distinct mass patterns. The Ball Mapper framework addresses these limitations by being parameter-efficient controlled mainly by distance parameters (ϵ, δ) selected via data-driven strategies and by adapting its cover to the data structure, eliminating arbitrary tiling. Crucially, spectral information is vectorized: each feature vector encodes a discrete mass spectrum, thus *m*/*z* composition drives clustering. This enables grouping by chemical similarity as well as retention proximity, allowing the method to distinguish coeluting compounds or isolate unique *m*/*z* signatures that segmentation approaches might overlook.

Overall, the integrated Ball Mapper approach offers a complementary perspective to classical chemometrics. It excels at detecting local features and patterns (e.g., a family of terpenes present only in one wine region) while still summarizing global structure (sample clusters by origin) in a single framework. Notably, by repeating the Ball Mapper analysis over many bootstrap iterations and constructing consensus clusters (RGs), we introduced a level of statistical validation and stability assessment rarely present in PCA or tile-based methods. The Algorithm 1 ensured that the groupings we discuss (Figures 4 and 5) are reproducible features of the data, not artifacts of a particular data split or noise realization. This robustness is a distinct advantage in complex chemical data sets where spurious patterns can arise. The end result is a topologically informed map that mirrors authentic relationships in the data (for example, grouping wines by winery or region) which strengthens confidence in the chemometric conclusions.

#### 4.2.6 Chemical Significance of ruMS Vectors in Context of Wines

A central outcome of this study is the demonstration that ruMS vectors can be used as chemically interpretable and statistically discriminative descriptors of complex samples. Unlike principal components or latent variables, each ruMS corresponds to a concrete set of ions (*m*/*z* values) from a chromatographic feature that can associate with a particular compound or class of compounds. This gives analysis a high degree of chemical transparency. For instance, rather than simply stating that “PC1 separates French and Tokaj wines,” now it is possible to pinpoint specific ruMS vectors (e.g., a furanone lactone and a methylthio-propanol) that drive this separation and relate them to known enological characteristics. The ruMS vectors identified by Ball Mapper effectively bridge multivariate statistics and chemical knowledge. Many of the discriminative ruMS correlate with known compound classes and regional wine origins: e.g., terpene-derived odorants were predominantly found in the Tokaj wines, whereas sulfur-containing ruMS vectors (like methylthio-propanol) were more characteristic of Sauternes, reflecting differences in grape variety and Botrytis strain metabolism between regions. Likewise, higher levels of phenolic ruMS (such as oak lactones and phenol derivatives) were noted in Tokaj region samples that undergo extensive barrel aging, versus higher alcohol/aldehyde ruMS in Sauternes which can result from different fermentation dynamics.

The utility of ruMS vectors for wine classification is clear in their ability to separate wines by country and even producer. Many ruMS were unique to one origin, acting as “fingerprint” compounds linked to terroir and vinification. For instance, C_6_–C_10_ ethyl esters strongly indicated Tokaj wines (enriched by the putňa process), while specific thioethers or lactones pointed to Sauternes (from *Botrytis cinerea*). Representative groups of wines from ruMS vectors revealed not only which features differ but also how they shape aroma profiles reflecting origin. This is valuable for authenticity studies, as it allows partial ruMS annotations to be informative without full compound identification. The results presented here demonstrate that even tentative assignments, such as a ruMS feature identified as a terpene or furan, provide insight when combined with uniqueness and statistical relevance.

Finally, it is important to acknowledge that a considerable fraction of features in these rich data sets remain unidentified by spectral libraries. This highlights both a challenge and an opportunity. The challenge is that untargeted analysis of complex natural products (such as noble rot wines) will inevitably encounter novel or poorly cataloged compounds, and full identification of all characteristics is impractical. The opportunity lies in the fact that ruMS profiling allow us to still extract value from these unknowns. By classifying unknown ruMS by their discrete mass spectral fragmentation patterns and retention characteristics, it was possible to group them into plausible chemical families (terpenoids, norisoprenoids, medium-chain acids, etc.). These groupings help formulate hypotheses about how botrytization or regional winemaking traditions influence the VOC profile of the wine. Thus, even without full identification, ruMS features contribute to a deeper mechanistic understanding of the chemical terroir. Going forward, the most informative of these markers (for example, a Tokaj-specific unknown furanone or a Sauternes-specific sulfur compound) can be prioritized for targeted structure elucidation or synthesis. This data-driven selection of such features can greatly focus future studies, reducing the analytical burden by highlighting a short list of high-impact compounds.

It revealed stable sample clusters reflecting real-world categories, similar to PCA or PLS-DA, while preserving chemical detail. At the same time, it identified key spectral features driving these differences, as in targeted biomarker analysis, but through an unsupervised, discovery-based approach. This dual outcome exemplifies how the proposed algorithm can complement traditional chemometric strategies: by preserving the integrity of the chemical information (rather than compressing it into abstract variables), it was possible to obtain results that a chemist can directly interpret and validate. The ruMS vectors identified here not only discriminate the wines statistically but also map onto known VOC classes linked to grape variety, fermentation, aging, and regional terroir.

## 5 Conclusions

We developed a structured workflow to apply Ball Mapper to high-dimensional spectral data. Raw mass spectra were transformed into normalized vectors (nMS), from which representative spectral vectors (rMS) were extracted using Ball Mapper. Relatively unique spectral vectors (ruMS) were then identified as points geometrically distant from other samples, capturing sample-specific traits. To assess reproducibility, frequency matrices from randomized Ball Mapper views quantified how consistently ruMS vectors occupied regions in high-dimensional space. These matrices enabled hierarchical clustering and the identification of representative groups (RGs) of samples sharing stable topological features. This dual-level interpretation—at both feature and sample levels—provides a robust, untargeted, and noise-tolerant framework for GC × GC–HR-TOF-MS data. Although demonstrated on wines, the approach is general and applicable to any chemically diverse system with chromatographic mass spectral data, enabling chemists to link spectral structure with metadata in a unified framework.

Ball Mapper further enabled data-driven selection of informative compounds for targeted analysis, reducing computational cost while retaining chemical relevance. The framework complements classical chemometric tools by revealing patterns not captured by PCA or PLS-DA and is broadly applicable to environmental, food, and biological mixtures. Even partial feature annotation, when guided by this statistical–topological approach, can yield valuable insights into complex chemical systems. Overall, our results demonstrate that integrating TDA with analytical chemistry complements established methods and offers deeper understanding of chemical diversity. In wine chemistry and beyond, this synergy of topology, statistics, and chemistry provides a powerful route to disentangle complexity and extract meaning from large analytical data sets.

## Supplementary Material





## Notes

It presents a total of 2792 *m*/*z* distributions obtained from GC × GC with HR-TOF-MS chromatograms of botrytized wine samples; Characteristic features were identified using raw retention time data in both dimensions and corresponding mass spectra; Unique retention times were compared with ChromaTOF-processed data to confirm feature identity; In total, 1191 features were matched to compounds, with 239 assigned to specific VOC classes, reflecting the full chemical complexity of the analyzed samples; The HR-TOF-MS detection was conducted at a data acquisition rate of 100 spectra per second, yielding more than 470,000 mass spectral distributions; Because raw data were utilized and each 2D peak occupies a finite area in both the first and second chromatographic dimensions, ruMS signals appear multiple times within a single sample; This repetition arises because the same mass spectral distribution is associated not with a single point but with the entire surface of the 2D peak; Chemical labels were assigned through EI spectral matching in combination with retention index data, indicating that highly similar spectra within the same chromatographic region may correspond to the same compound (XLSX)
It lists 334 identified compounds from the set of 1191 features, assigned to known VOC classes, including compounds occurring in multiple samples; Additionally, 46 distinct compounds were tentatively classified based on VOC class, although their exact identities could not be confirmed (XLSX)

## References

[1] Ochoa G. S., Sudol P. E., Trinklein T. J., Synovec R. E. (2022). Class comparison
enabled mass spectrum purification for comprehensive two-dimensional
gas chromatography with time-of-flight mass spectrometry. Talanta.

[2] Schoneich S., Cain C. N., Freye C. E., Synovec R. E. (2022). Optimization of
parameters for ROI data compression for nontargeted analyses using
LC–HRMS. Anal. Chem..

[3] Koljančić N., Onça L., Khvalbota L., Vyviurska O., Gomes A. A., Špánik I. (2024). Region of
interest
selection in heterogeneous digital image: Wine age prediction by comprehensive
two-dimensional gas chromatography. Curr. Res.
Food Sci..

[4] Dłotko, P. Ball mapper: a shape summary for topological data analysis. arXiv:1901.07410. arXiv.org e-Print archive. https://arxiv.org/abs/1901.07410. 2019.

[5] Dłotko P., Gurnari D., Sazdanovic R. (2024). Mapper–Type Algorithms for
Complex Data and Relations. J. Comput. Graphical
Stat..

[6] Vyviurska O., Koljančić N., Thai H. A., Gorovenko R., Špánik I. (2021). Classification
of botrytized wines based on producing
technology using flow-modulated comprehensive two-dimensional gas
chromatography. Foods.

[7] Caratti A., Squara S., Bicchi C., Tao Q., Geschwender D., Reichenbach S. E., Ferrero F., Borreani G., Cordero C. (2023). Augmented
visualization by computer vision and chromatographic fingerprinting
on comprehensive two-dimensional gas chromatographic patterns: Unraveling
diagnostic signatures in food volatilome. J.
Chromatogr. A.

[8] Mondello L., Cordero C., Janssen H.-G., Synovec R. E., Zoccali M., Tranchida P. Q. (2025). Comprehensive two-dimensional gas
chromatography–mass
spectrometry. Nat. Rev. Methods Primers.

[9] Reichenbach S. E., Tian X., Cordero C., Tao Q. (2012). Features for
non-targeted
cross-sample analysis with comprehensive two-dimensional chromatography. J. Chromatogr. A.

[10] Stilo F., Bicchi C., Jimenez-Carvelo A. M., Cuadros-Rodriguez L., Reichenbach S. E., Cordero C. (2021). Chromatographic fingerprinting
by
comprehensive two-dimensional chromatography: Fundamentals and tools. TrAC, Trends Anal. Chem..

[11] Marney L. C., Siegler W. C., Parsons B. A., Hoggard J. C., Wright B. W., Synovec R. E. (2013). Tile-based Fisher-ratio
software for improved feature
selection analysis of comprehensive two-dimensional gas chromatography–time-of-flight
mass spectrometry data. Talanta.

[12] Parsons B. A., Marney L. C., Siegler W. C., Hoggard J. C., Wright B. W., Synovec R. E. (2015). Tile-based Fisher
ratio analysis of comprehensive two-dimensional
gas chromatography time-of-flight mass spectrometry (GC × GC–TOFMS)
data using a null distribution approach. Anal.
Chem..

[13] Watson N.
E., Parsons B. A., Synovec R. E. (2016). Performance evaluation of tile-based
Fisher Ratio analysis using a benchmark yeast metabolome dataset. J. Chromatogr. A.

[14] Sudol P. E., Ochoa G. S., Synovec R. E. (2021). Investigation of
the limit of discovery
using tile-based Fisher ratio analysis with comprehensive two-dimensional
gas chromatography time-of-flight mass spectrometry. J. Chromatogr. A.

[15] Cain C. N., Trinklein T. J., Ochoa G. S., Synovec R. E. (2022). Tile-based
pairwise
analysis of GC× GC-TOFMS data to facilitate analyte discovery
and mass spectrum purification. Anal. Chem..

[16] Reichenbach S. E., Zini C. A., Nicolli K. P., Welke J. E., Cordero C., Tao Q. (2019). Benchmarking machine
learning methods for comprehensive chemical
fingerprinting and pattern recognition. J. Chromatogr.
A.

[17] Pérez-Cova M., Platikanov S., Tauler R., Jaumot J. (2022). Quantification strategies
for two-dimensional liquid chromatography datasets using regions of
interest and multivariate curve resolution approaches. Talanta.

[18] Brilhante N. S., Bizzo H. R., Caratti A., Squara S., Cordero C. (2024). Artificial
intelligence tools and concepts give access to authenticity and quality
information in Brazilian olive oil volatilome. J. Food Compos. Anal..

[19] Derwae H., Nijs M., Geysels A., Waelkens E., De Moor B. (2023). Spatiochemical
characterization of the pancreas using mass spectrometry imaging and
topological data analysis. Anal. Chem..

[20] Singh, G. ; Mémoli, F. ; Carlsson, G. In Eurographics Symposium on Point-Based Graphics; Botsch, M. ; Pajarola, R. ; Chen, B. ; Zwicker, M. , Eds.; The Eurographics Association, 2007.

[21] McInnes L., Healy J., Saul N., Melville J. (2018). UMAP: Uniform Manifold
Approximation and Projection. J. Open Source
Software.

[22] Klaila G., Vutov V., Stefanou A. (2023). Supervised topological
data analysis
for MALDI mass spectrometry imaging applications. BMC Bioinf..

[23] Offroy M., Duponchel L. (2016). Topological
data analysis: A promising big data exploration
tool in biology, analytical chemistry and physical chemistry. Anal. Chim. Acta.

[24] Duponchel L. (2018). Exploring
hyperspectral imaging data sets with topological data analysis. Anal. Chim. Acta.

[25] Lukmanov R. A., Riedo A., Wacey D., Ligterink N. F., Grimaudo V., Tulej M., de Koning C., Neubeck A., Wurz P. (2021). On topological analysis of fs-LIMS
data: Implications for in situ planetary mass spectrometry. Front. Artif. Intell..

[26] Ruiz-Perez D., Guan H., Madhivanan P., Mathee K., Narasimhan G. (2020). So you think
you can PLS-DA?. BMC Bioinf..

[27] Freye C. E., Bowden P. R., Greenfield M. T., Tappan B. C. (2020). Non-targeted discovery-based
analysis for gas chromatography with mass spectrometry: A comparison
of peak table, tile, and pixel-based Fisher ratio analysis. Talanta.

[28] Madukpe V. N., Zulkepli N. F. S., Noorani M. S. M., Gobithaasan R. (2025). Comparative
analysis of Ball Mapper and conventional Mapper in investigating air
pollutants’ behavior. Environ. Monit.
Assess..

[29] Akuaka G. O., Haris H., Madukpe V. N., Zarkasi K. Z., Furusawa G., Abdul Hamid B. A. (2025). Visualization
of physicochemical parameters’
behavior in leachate, baseliner, and surface water during dry and
rainy seasons at a sanitary landfill. Environ.
Monit. Assess..

[30] Park S. A., Kim Y., Gurnari D., Dłotko P., Hahn J. (2025). Spatial Analysis of
Malignant-Immune Cell Interactions in the Tumor Microenvironment Using
Topological Data Analysis. bioRxiv.

[31] Koljančić N., Gomes A. A., Špánik I. (2023). A non-target geographical
origin screening of botrytized wines through comprehensive two-dimensional
gas chromatography coupled with high-resolution mass spectrometry. J. Sep. Sci..

[32] Furdíková K., Machyňáková A., Drtilová T., Špánik I. (2020). Comparison of different
categories of Slovak Tokaj
wines in terms of profiles of volatile organic compounds. Molecules.

[33] Edelsbrunner, H. ; Harer, J. Computational Topology: An Introduction; American Mathematical Society, 2010.

[34] Carlsson G. (2009). Topology and
data. Bull. Am. Math. Soc..

[35] Madukpe, V. N. ; Ugoala, B. C. ; Zulkepli, N. F. S. A Comprehensive Review of the Mapper Algorithm, a Topological Data Analysis Technique, and Its Applications Across Various Fields (2007–2025). arXiv:2504.09042. arXiv.org e-Print archive. https://arxiv.org/abs/2504.09042. 2025.

[36] pyBallMapper: A Python Implementation of Ball Mapper. https://pypi.org/project/pyBallMapper/, (accessed Oct 14, 2023).

[37] Hubert L., Arabie P. (1985). Comparing partitions. J.Classif..

[38] Cieslak M. C., Castelfranco A. M., Roncalli V., Lenz P. H., Hartline D. K. (2020). t-Distributed
Stochastic Neighbor Embedding (t-SNE): A tool for eco-physiological
transcriptomic analysis. Mar. Genomics.

[39] Spillman P. J., Pollnitz A. P., Liacopoulos D., Pardon K. H., Sefton M. A. (1998). Formation
and degradation of furfuryl alcohol, 5-methylfurfuryl alcohol, vanillyl
alcohol, and their ethyl ethers in barrel-aged wines. J. Agric. Food Chem..

[40] Vanderhaegen B., Neven H., Daenen L., Verstrepen K. J., Verachtert H., Derdelinckx G. (2004). Furfuryl ethyl ether: Important aging
flavor and a new marker for the storage conditions of beer. J. Agric. Food Chem..

[41] Correia A. C., Miljić U., Jordão A. M. (2023). Storage of a white wine with different
untoasted wood species: Impact on the chemical composition and sensory
characteristics. Eur. Food Res. Technol..

[42] Oberholster A., Wen Y., Dominguez
Suarez S., Erdmann J., Cauduro
Girardello R., Rumbaugh A., Neupane B., Brenneman C., Cantu A., Heymann H. (2022). Investigation of different winemaking
protocols to mitigate smoke taint character in wine. Molecules.

[43] Schreier P., Jennings W. G. (1979). Flavor composition
of wines: a review. Crit. Rev. Food Sci. Nutr..

[44] Garde-Cerdán T., Lorenzo C., Carot J., Esteve M., Climent M., Salinas M. (2009). Differentiation of
barrel-aged wines according to their
origin, variety, storage time and enological parameters using fermentation
products. Food Control.

[45] Lorenzo C., Garde-Cerdán T., Pedroza M. A., Alonso G. L., Salinas M. R. (2009). Determination
of fermentative volatile compounds in aged red wines by near infrared
spectroscopy. Food Res. Int..

[46] Xu J., Wu H., Wang Z., Zheng F., Lu X., Li Z., Ren Q. (2018). Microbial
dynamics and metabolite changes in Chinese Rice Wine fermentation
from sorghum with different tannin content. Sci. Rep..

[47] Moreira N., Mendes F., Pereira O., De Pinho P. G., Hogg T., Vasconcelos I. (2002). Volatile sulphur
compounds in wines related to yeast
metabolism and nitrogen composition of grape musts. Anal. Chim. Acta.

[48] Barbe J.-C., de Revel G., Joyeux A., Lonvaud-Funel A., Bertrand A. (2000). Role of carbonyl compounds in SO2
binding phenomena
in musts and wines from botrytized grapes. J.
Agric. Food Chem..

[49] Ma Y., Wu H., Liu Q., Wang L., Dou X., Yang L., Yang J. (2016). Study on the Aromatic Components of Green Plum Wine by HS-SPME-GC-MS. Asian Agric. Res..

[50] Miklósy É., Kerényi Z. (2004). Comparison of the volatile aroma
components in noble rotted grape berries from two different locations
of the Tokaj wine district in Hungary. Anal.
Chim. Acta.

[51] Lan W., Cheng W., Li R., Zhang M., Li M., Zhang Y., Zhou Y. (2024). Comparison
of Flavor Differences
between the Juices and Wines of Four Strawberry Cultivars Using Two-Dimensional
Gas Chromatography-Time-of-Flight Mass Spectrometry and Sensory Evaluation. Molecules.

